# Pregnancy from a Legal Standpoint—II

**Published:** 1909-02-27

**Authors:** 


### Medico-Legal Points.

### PREGNANCY FROM A LEGAL STANDPOINT—II.

If medical evidence as to the existence of pregnancy is called, the jury, it has been ruled, must return into Court, and hear such testimony given publicly upon oath. In strictness, the question to be decided is not whether the prisoner is or is not *priviment ensient*—*i.e.* barely with child: having conceived, "but not with quick child"—but whether or not she is *ensient de vive enfant*; but in practice, a jury of women will lean to mercy's side; for, says Hale, "I have rarely found but the compassion of their sex is gentle to them in their verdict, if there being any colour to support a sparing verdict." In the *Norwich Case*, however, a jury of matrons negatived the plea, though the woman was in the fifth month of her pregnancy.

In *Wycherley's Case*, Gurney, B., directed the surgeon (Mr. Greatorex) in these words: "'Quick with child' is having conceived. 'With quick child' is when the child has quickened. Do you understand the distinction I make?" It would seem that he ought not to have used "quick," but "barely," in the first sentence. But in truth the language of the procedure, and the very procedure itself, are founded upon a physiological error long since rejected by scientific opinion. "The vulgar opinion," says Taylor (4th edition, vol. ii., p. 161), "is that the fœtus only receives life when the woman quickens. As ovum, embryo, or fœtus, however, the contents of the uterus are as much endowed with special and independent vitality in the earlier as in the later periods of gestation." That obscure change which, through error, is popularly known as "quickening," is nothing whatever to the purpose when the question is whether an individual existence can be attributed at the time to the child; yet the law has made this change the criterion whereby a jury of mothers must determine if the unborn offspring can be regarded as having a separate life, and so separate rights of its own. The writers on medical jurisprudence, without exception, condemn both the procedure and the false physiological theory underlying it. In practice, pregnancy (when satisfactorily established) has been accepted as justifying a favourable verdict.

Supposing a verdict of "with quick child" to be returned, the prisoner, as a matter of right and *ex necessitate legis*, is entitled to be reprieved. This reprieve is granted, not for the woman's own sake, but in favour of the offspring. And it holds good, at all events, until either the woman shall have been delivered or proved by the course of nature not to have been with child. Though the prisoner may not have been pregnant in the first instance, she may in the meantime find herself in a state of real *ensienture*, and she may none the less plead her state as a ground of respite. But it has been said that if she has once, under reprieve, been delivered of a child, and afterwards become with child again, the plea of pregnancy will not avail her again, and the sentence of the law must proceed, regardless of her state, whether she be with quick child or not (Staindford P. del C. 198), and "the gaoler shall be punished for not looking better to her" (2 Hale 413), the ground being that a woman ought not to be able to evade or postpone execution by her own incontinence and voluntary act.

The French law (Penal Code Act 217) appears to be satisfied by medical proof of pregnancy. The New York Statutes provide that the question of a female convict s pregnancy shall, in a capital case, be decided by a jury of six qualified medical men.

A jury of matrons may also be called into requisition in civil cases. An inquisition by a jury of matrons is the appointed means of deciding under a writ *de ventre inspiciendo*, first, whether the woman named therein is with child or not, and, if she be found with child, secondly, at or about what date her delivery is to be expected. The writ is obtained by petition from the Court of Chancery, and is addressed to the Sheriff; and after reciting an allegation of pregnancy and a possibility of fraud, it bids the Sheriff take with him certain *discreti et legales milites et discretae et legales mulieres* (twelve of each became ultimately the fixed number) and visit the lady named in the writ, and cause the *mulieres* to use their best endeavours to ascertain whether the allegation be true; if they find it true, they are also to state when the birth is likely to take place. If the jury of matrons have found pregnancy, a second writ issues providing that the woman shall be guarded closely *in castello* during the period of her pregnancy.

A woman may assert that she is pregnant at the time of her husband's death, and the heir-at-law may obtain a writ to require some proof of her alleged pregnancy, as his right to the estate of which her husband died possessed may be materially affected by the result.

### Medical Examination of Servants.

Mistresses frequently accuse their servants of being pregnant, or of suffering from some form of venereal disease, and send for a doctor for examination purposes. Under these circumstances the doctor has no right whatever to examine the servant unless the latter fully, freely, and willingly gives her consent, and he should not proceed with such examination until her consent has been personally given to him. Moreover, unless the servant expressly permits it, the doctor has no right to communicate the opinion resultant on the examination to the mistress. Mere compliance, it must be clearly understood, does not mean consent. The following case from Taylor's "Medical Jurisprudence" (5 edit. vol. 1, p. 67) is a warning to all practitioners who are consulted in such cases:

The plaintiff was a domestic servant in the employ of two of the defendants. On the return of her mistress from a visit, the latter was told by a charwoman, whom she employed, that the plaintiff was in the family way. She at once ordered the plaintiff to pack up and be ready to leave after the doctor, who had been sent for, had been and examined her. The defendant examined her and found that she was not pregnant. The evidence as to consent was conflicting, the plaintiff stating on oath that she did not consent, the defendant stating on oath as positively that she did. Lindley, J., withdrew the case from the jury as to master and mistress, but left the case as to the surgeon to the jury, who found a verdict for the defendant. The case was heard again on appeal, when the two judges, Lopes and Lindley, differed diametrically on this point of consent. The former observed that the plaintiff protested against the examination, and he did not see what more she could have done. Lindley, J., observed that the plaintiff was not a child, being twenty-eight years old, and could easily have prevented the examination if she wished. The verdict of the jury was upheld. The surgeon examined the plaintiff without any witness, and without her consent having been expressly given, though the evidence on this point was conflicting. The case was carried from Court to Court, and though the defendants won all through, they were subjected to heavy costs as well as much annoyance. Lord Esher, one of the Lords of Appeal, condemned strongly the practice of sending for a doctor to examine a servant to see if she was pregnant. He urged that masters and mistresses ought not to regard such a circumstance, even if true, as an insult to themselves, and that their aim should be to get the servant away quietly without any exposure.

